# Hematopoietic Pyk2 regulates migration of differentiated HL-60 cells

**DOI:** 10.1186/1476-9255-7-26

**Published:** 2010-05-27

**Authors:** Lin Wang, Jonathan Learoyd, Yingli Duan, Alan R Leff, Xiangdong Zhu

**Affiliations:** 1 Department of Medicine, The University of Chicago, 5841 S Maryland Avenue, Chicago, IL 60637, USA; 2 Department of Neurobiology, The University of Chicago, 5841 S Maryland Avenue, Chicago, IL 60637, USA; 3 Department of Pharmacology and Physiology, The University of Chicago, 5841 S Maryland Avenue, Chicago, IL 60637, USA; 4 Department of Pediatrics, The University of Chicago, 5841 S Maryland Avenue, Chicago, IL 60637, USA; 5 Department of Anesthesia and Critical Care, The University of Chicago, 5841 S Maryland Avenue, Chicago, IL 60637, USA; 6 Committees on Clinical Pharmacology and pharmcogenomics, The University of Chicago, 5841 S Maryland Avenue, Chicago, IL 60637, USA; 7 Department of Cell Physiology and Molecular Medicine, The University of Chicago, 5841 S Maryland Avenue, Chicago, IL 60637, USA; 8 Department of Pediatrics, China-Japan Friendship Hospital, 2 East Yinghuayuan Street, Beijing, 100029, China

## Abstract

**Background:**

Pyk2 is a non-receptor cytoplasmic tyrosine kinase that belongs to the focal adhesion kinase family and has been implicated in neutrophil spreading and respiratory burst activity caused by TNF-α. However, the role of Pyk2 in neutrophil migration is incompletely defined. In this study, we tested the hypothesis that Pyk2 regulates the migration of neutrophil-like differentiated HL-60 cells subsequent to β2-integrin mediated cell adhesion.

**Methods:**

HL-60 cells were induced to differentiate into neutrophil-like cells (dHL60) by incubation in medium containing 1.25% DMSO for up to 4 days. Pyk2 expression and tyrosine phosphorylation was measured by Western blot analysis. Adhesion of dHL60 cells to plated fibrinogen was measured by residual myeloperoxidase activity. dHL60 cell migration was evaluated using a 96-well chemoTx chamber.

**Results:**

Western blot analysis demonstrated that hematopoietic Pyk2 was predominantly expressed after HL60 cell differentiation. Pyk2 was tyrosine phosphorylated upon adhesion of dHL60 cells to plated fibrinogen in the presence of fMLP. By contrast, tyrosine phosphorylation of Pyk2 was insignificant in dHL60 cells treated in suspension with fMLP. Antibodies against CD18 blocked both phosphorylation of Pyk2 and adhesion of dHL60 cells to fibrinogen, demonstrating that phosphorylation of Pyk2 was β_2_-integrin dependent. TAT-Pyk2-CT, a dominant negative fusion protein in which the TAT protein transduction domain was fused to the c-terminal Pyk2, attenuated fMLP-stimulated spreading, migration and phosphorylation of endogenous Pyk2 without blocking adhesion of dHL-60 cells to fibrinogen. Similarly, silencing of Pyk2 expression by siRNA in dHL60 cells also attenuated dHL60 cell migration caused by fMLP. Phospho-Pyk2 was evenly distributed around cell membrane circumferentially in unstimulated dHL-60 cells adherent to plated fibrinogen. In dHL60 cells treated with fMLP to cause cell spreading and polarization, Pyk2 was concentrated at the leading edge of pseudopods or at the trailing edge of uropods during migration of neutrophilic dHL-60 cells.

**Conclusions:**

We conclude that Pyk2 is activated by β2-integrin adhesion. The activated concentration of Pyk2 and colocalization with F-actin in pseudopodia suggests that Pyk2 may regulate cell spreading and migration in dHL60 cells.

## Background

Polymorphonuclear neutrophils (PMNs) play a central role in the acute inflammatory response and are also closely associated with tissue injury [1]. Full activation of neutrophils by a soluble inflammatory stimulus requires a co-stimulatory signal initiated by integrin binding to endothelial cells or extracellular matrix proteins [2,3]. Integrins fix cellular protrusions to extracellular matrix proteins, interact with the intracellular actin cytoskeleton, and trigger the association of many different signaling proteins at focal contacts [4].

Proline-rich tyrosine kinase 2 (Pyk2), also known as cell adhesion kinase β, is a non-receptor cytoplasmic tyrosine kinase that belongs to the focal adhesion kinase family [5]. Focal adhesion kinases are responsible for transferring signals from integrins to downstream signaling cascades that regulate cell growth and migration in adherent cells [6,7]. Pyk2 is expressed abundantly in hematopoietic cells and neural cells [8,9]. Human neutrophils express both FAK and Pyk2, but only Pyk2 appears to regulate neutrophil function [10,11]. Previous studies have identified Pyk2 in human neutrophils, localized it to focal adhesion-like structures, and demonstrated its association with paxillin during stimulation of adherent neutrophils by TNFα [12]. However, the role of Pyk2 in neutrophil migration is incompletely defined.

Differentiated HL60 cells are commonly used as a model system for neutrophil migration [13]. Human blood neutrophils have a short half-life *in vitro *and are terminally differentiated. Genetic modification of Pyk2 expression in mature cells such as neutrophils using current techniques has been largely unsuccessful. Therefore in this study, we chose the differentiated HL60 cells as a model for human neutrophils to study the role of Pyk2 in neutrophil migration. In these studies, we found that the hematopoietic isoform of Pyk2 is predominantly expressed in dimethyl sulfoxide (DMSO)-differentiated HL-60 (dHL60) cells. Stimulation of dHL60 cells with chemotactic peptide formyl-Met-Leu-Phe (fMLP) induced tyrosine phosphorylation of Pyk2 subsequent to β2 integrin adhesion. Using transduction of TAT-conjugated Pyk2-derived C-terminal protein (amino acid 680-1009) as a specific inhibitor, we demonstrated that Pyk2 inhibition blocked significantly fMLP-induced migration without blocking the ability of dHL60 cells to adhere to plated fibrinogen. Phospho-Pyk2 was co-localized with F-actin, mainly at the leading edge of lamellipodia in migrating dHL-60 cells adherent to plated fibrinogen. Our data indicate that Pyk2 is activated upon β2-integrin binding to fibrinogen and likely facilitates cell spreading and migration by co-localizing with cytoskeletal structures in response to chemoattractants.

## Methods

### Materials

HL-60 cells and RPMI 1640 medium were obtained from American Type Culture Collection (Manassas, VA). Fetal bovine serum (FBS) was purchased from Hyclone (Logan, UT). L-glutamine was obtained from Invitrogen (Eugene, OR). Fibrinogen (Fg), dimethyl sulfoxide (DMSO) and formyl-Met-Leu-Phe (fMLP) were obtained from Sigma-Aldrich (St. Louis, MO). The primary antibodies utilized in this study include anti-Pyk2, anti-tyrosine 402 phospho-Pyk2 (Cell Signaling, MA), mouse IgG (Southern biotech, UT), and anti-CD18 mAb (7E4, Ancell, MN). The secondary antibodies include horseradish peroxide conjugated anti-mouse and anti-rabbit antibodies from Amersham (Arlington Heights, IL), BODIPY FL goat anti-rabbit IgG, Alexa Fluor 594 goat anti-mouse IgG (H+L) and Alexa Fluor 647 phalloidin from Invitrogen Molecular Probes (Eugene, OR). 96-well microplates for adhesion assay were purchased from Costar (Corning, NY). Migration assay microplates were purchased from Neuro Probe (Gaitherberg, MD). TAT-Pyk2-CT was produced in our laboratory as described previously [14,15].

### HL-60 Cell culture and differentiation

HL-60 cells were cultured in RPMI-1640 medium supplemented with 10% fetal bovine serum, 400 mM L-glutamine, 50 μM gentamycin, 25 mM HEPES, 2 g/L sodium bicarbonate, 1 mM sodium pyruvate in a humid atmosphere of 5% CO_2 _at 37°C. For differentiation into a neutrophilic phenotype, HL-60 cells were resuspended in fresh medium at a concentration of 10^5^/ml and then differentiated with 1.25% (v/v) DMSO for up to 9 days [16]. Cell viability was assessed by exclusion of 0.2% trypan blue and was routinely >90% before and after differentiation. Assessment of neutrophilic differentiation was performed on days 0, 3, 6, 9 by several criteria including 1) morphological change as nuclear segmentation and granules using May-Grunwald Giemsa staining, 2) CD11b expression detected by flow cytometry and 3) responsiveness to PAF, fMLP or TNF-α stimulated adhesion to fibrinogen-coated plates. dHL60 cells that were differentiated for 4-6 days were used for adhesion and migration assays.

### Adhesion assay

dHL60 cell adhesion was assessed as the residual myeloperoxidase (MPO) activity of adherent cells [17,18]. 96-well microplates were coated with 100 μg/ml Fg overnight at 4°C [19]. dHL60 cells (10^5^) in HBSS containing 0.2% BSA were seeded on to each well of a microplate with or without 10^-6 ^M fMLP in a total volume of 100 μl for 30 min at 37°C. After incubation, the reaction wells were washed with HBSS 3 times. Serial dilutions of the original cell suspension were added to the empty wells to generate the standard curve. 100 μl of the myeloperoxidase substrate (0.01% H_2_O_2_, 0.167 mg/mL *O*-dianisidine dihydrochloride and 0.5% hexadecyltrimethylammonium bromide in 50 mM potassium phosphate buffer, pH 5.5) was added to each well, and the reaction was terminated with 4N sulphuric acid. Absorbance was then measured at 405 nm in a microplate reader (Molecular devices, Sunnyvale, CA). The adherent cells were expressed as percentage of the total cells added.

The blocking effect of mouse monoclonal antibodies (mAb) against the common β chain of β2 integrin, anti-CD18 (clone 7E4), was tested by preincubation of 10 μg/ml antibodies with dHL60 cells for 30 min at 4°C before adding to the 96-well microplates. Similarly, the inhibitory effect of TAT-Pyk2-CT on adhesion of dHL60 cells to plated Fg was assessed after 30 min preincubation at 37°C.

### Migration assay

dHL60 cell migration was evaluated using a 96-well chemoTx chamber as described previously [15,20]. dHL60 cells were pretreated with TAT-Pyk2-CT or TAT-GFP control as above. dHL60 cells then were added to the upper chambers of ChemoTx 96-well transwell plates with 5 μm pore filters and allowed to migrate for 90 min at 37°C in 5% CO_2 _towards the lower chamber containing 10^-8 ^M fMLP. Migrated cells from lower chamber were centrifuged at 400 g for 10 min and collected at the bottom of the lower chambers. As described for the adhesion assay, MPO activity in the migrated cells was quantitated against a standard curve of diluted cells, and the result was expressed as a percentage of cells added to the top of the plate.

### Spreading assay

dHL60 cells were pre-treated with TAT-Pyk2-CT and TAT-GFP as above. Cells were then added to fibrinogen (Fg)-coated 8-well chamber slides containing 10^-6 ^M fMLP and incubated for 30 min at 37°C in 5% CO_2_. Cells were viewed through a phase-contrast microscope, and selected fields typical of each slide were photographed for analysis.

### Western blot analysis

dHL60 cell lysates were prepared by boiling with SDS sample buffer for 5 min. Whole cell lysates were separated by 10% SDS-PAGE and transferred to polyvinylidene fluoride membranes. The membranes were blocked with 2% bovine serum albumin (BSA) in Tris-buffered saline plus 0.1% Tween 20 (TBS/T) for 60 min at 37°C. This was followed by incubation with primary antibodies (1:1000) in TBS/T overnight at 4°C. The membrane was then washed and incubated with horseradish peroxidase conjugated anti-rabbit or mouse secondary antibodies (1:3000) in TBS/T for 60 min. After washing with TBS/T 3 times, enhanced chemiluminescence (ECL) assays were performed to visualize bands on X-ray films.

### Confocal microscopy

dHL60 cells (2 × 10^5^) were preincubated with TAT-Pyk2-CT or TAT-GFP control as above. Cells were plated onto 8-chamber glass slides coated with 100 μg/ml Fg and incubated with 10^-6 ^M fMLP at 37°C for 30 min. After fixation with 1% paraformaldehyde for 10 min, cells were permeabilized with 0.1% Triton-X100 in PBS for 4 min. The permeabilized cells then were incubated with Alexa Fluor 647-phalloidin for 60 min. To visualize the localization of phospho-Pyk2, cells were incubated with anti-phospho-Pyk2 antibody (1:40) diluted in PBS for 60 min and then incubated with Bodipy FL conjugated goat anti-rabbit IgG (1:1000) for another 60 min. After washing with PBS 3 times, cells were then analyzed with a laser-scanning confocal microscope imaging system (Leica SP2 AOBS, Mannheim, Germany). Photomicrographs were focused at the plane of cell contact with the substratum.

### Silencing of Pyk2 expression in dHL60 cells

A mixture of three separate siRNA duplexes targeting human Pyk2 were purchased from Dharmacon (Catalog #M-003165-03, Lafayette, CO). A single non-targeting scramble siRNA duplex (Catalog #D-001210-01) was used as control siRNA. HL60 cells were differentiated with 1.25% DMSO for 3 days, and 2 μM siRNA was then transfected into 4 × 10^6 ^cells in 100 μl of Nucleofector Solution V (Amaxa) using the T-019 program on the Nucleofector II machine (Amaxa), according to the manufacturer's recommendation (Lonza Walkerrsville Inc. Walkersville, MD). The cells then were recovered in medium with 1.25% DMSO and harvested 72 h later. The expression of Pyk2 after transfection was evaluated by Western blotting.

### Statistics

The results are expressed as Mean ± standard error of the mean. Data were analyzed using one-way ANOVA followed by Fisher's least significant difference method of post hoc analysis or paired t test; p < 0.05 was considered to be statistically significant.

## Results

### Neutrophilic differentiation and expression of Pyk2 in HL-60 cells

In preliminary studies, differentiation of HL60 cells to neutrophil-like cells was confirmed by 1) morphologic change including nuclear segmentation, 2) CD11b expression and 3) the ability to respond to PAF, TNFα or fMLP in adhesion and migration assays. For subsequent experiments requiring cell replication and characterization, dHL-60 cells thus were used to model human neutrophils.

**Figure 1:**
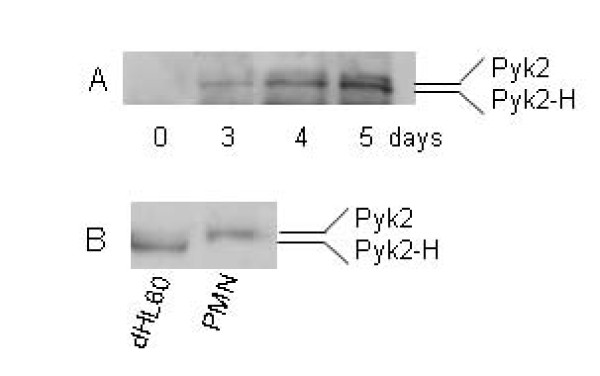
**Pyk2 expression in HL60 cells differentiated by DMSO**. A) HL60 cells were induced to differentiate toward neutrophil-like cells. HL60 cells were lysed before and 3 to 5 days after differentiation, and Pyk2 expression in these cells was analyzed by Western blot using anti-Pyk2 antibody. B) Pyk2 expression in dHL60 cells as compared with human blood neutrophils. Pyk2 expression was measured as above. Representative blot is shown (n = 3).

The predominant Pyk2 isoform differed slightly between human neutrophils and dHL60 cells. Pyk2 was not expressed in undifferentiated HL-60 cells. After differentiation for ≥ 3 days, two isoforms of Pyk2 were detected in dHL60 cells (Fig. 1A). The lower molecular weight (~105 kDa) Pyk2H was the predominant isoform expressed in dHL-60 cells. The full-length Pyk2 isoform (~110 kDa) was weakly expressed or absent in these cells. In contrast to dHL60 cells, mature human blood neutrophils expressed only the full-length Pyk2 isoform (Fig. 1B). There is no known functional difference between the two isoforms (see discussion).

### Tyrosine phosphorylation of Pyk2 is dependent on integrin-mediated cell adhesion

**Figure 2:**
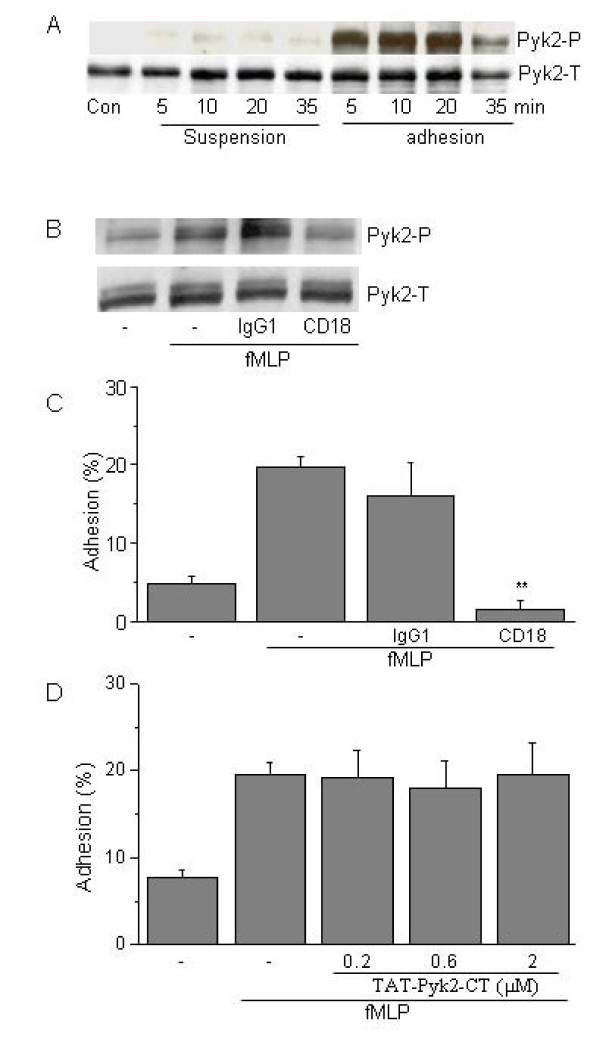
**Tyrosine phosphorylation of Pyk2 induced by fMLP in dHL-60 cells adhering to plated fibrinogen**. A) dHL-60 cells were either in suspension or added to plated fibrinogen, and then stimulated with 1 μM fMLP for indicated times at 37°C. Pyk2 activation was measured by its autophosphorylation detected by Y402 phosphorylation-specific antibody (upper panel). Total Pyk2 expression was measured by Western blot using anti-Pyk2 antibody (lower panel). B) Effect of anti-β2 integrin antibody on phosphorylation of Pyk2 induced by fMLP in adherent dHL60 cells. dHL60 cells were preincubated with 10 μg/ml antibody against CD18 for 30 min, and then added to Fg-coated plates with 1 μM fMLP for 10 min. Pyk2 tyrosine phosphorylation was then measured as above. C) Effect of anti-β2 integrin antibody on phosphorylation of Pyk2 induced by fMLP in adherent dHL60 cells. dHL60 cells were preincubated with 10 μg/ml anti-CD18 antibody for 30 min, and then added to Fg-coated plates with 1 μM fMLP for 30 min. Adhesion of dHL60 cells to plated Fg was then measured as residual myeloperoxidase assay. Results are presented as the mean ± SEM from 4 separate experiments. **p < 0.01 vs. fMLP alone, Fisher's LSD test. D) Effect of TAT-Pyk2-CT on adhesion of dHL-60 cells to plated fibrinogen. dHL-60 cells were preincubated with indicated concentrations of TAT-Pyk2-CT and then adhered to plated fibrinogen for 30 min in the presence of 1 μM fMLP. Adhesion of dHL-60 cells was measured by residual myeloperoxidase activity assay. Results are presented as the mean ± SEM from 4 separate experiments.

Adhesion to plated Fg greatly augmented Pyk2 phosphorylation. Phosphorylation of Pyk2 was only weakly detected in dHL-60 cells suspended in buffer after activation 10^-6 ^M fMLP. By contrast, Pyk2 phosphorylation was substantially induced in fMLP-stimulated cells adherent to plated Fg (Fig. 2A). Phosphorylation of Pyk2 was observed within 5 min and was maximal at 20 min.

Both tyrosine phosphorylation of Pyk2 and adhesion of dHL60 cells to Fg-coated plates were dependent on adhesion to β_2_-integrin. Blockade by mAb directed against the common β chain of β2-integrin, CD18, substantially attenuated Pyk2 phosphorylation elicited by fMLP as demonstrated by Western blot analysis. Mouse IgG1 isotype control had no effect on Pyk2 phosphorylation caused by adhesion (Fig. 2B). Similarly, adhesion of dHL60 cells to plated Fg caused by fMLP was significantly reduced by preincubation with anti-CD18 mAb. Mouse IgG1 caused no blockade of fMLP-induced adhesion (Fig. 2C). Adhesion of dHL60 cells to Fg-coated plates increased from 4.9 ± 0.9% (negative control) to 19.6 ± 1.4% (fMLP-treated; p < 0.01). Preincubation with anti-CD18 mAb blocked fMLP stimulated adhesion to 1.4 ± 1.2% (p < 0.01 vs. fMLP-treated positive control).

### Effect of TAT-Pyk2-CT on adhesion of dHL60 cells to plated fibrinogen

Pyk2 had no effect in causing adhesion of dHL-60 cell elicited by fMLP. Adhesion of dHL60 cells to plated Fg increased from 7.7 ± 1.0% to 19.6 ± 1.4% after treatment with 10^-6 ^M fMLP for 30 min. Pretreatment with TAT-Pyk2-CT, a selective cell permeable dominant negative protein inhibitor of Pyk2 [10,15], did not block adhesion of dHL60 cells to plated Fg induced by fMLP (Fig. 2D). These results suggest that Pyk2 has no regulatory role in the induction of adhesion caused by the β_2_-integrin.

### Effect of Pyk2 inhibition on fMLP-induced transwell migration of dHL60 cells

**Figure 3:**
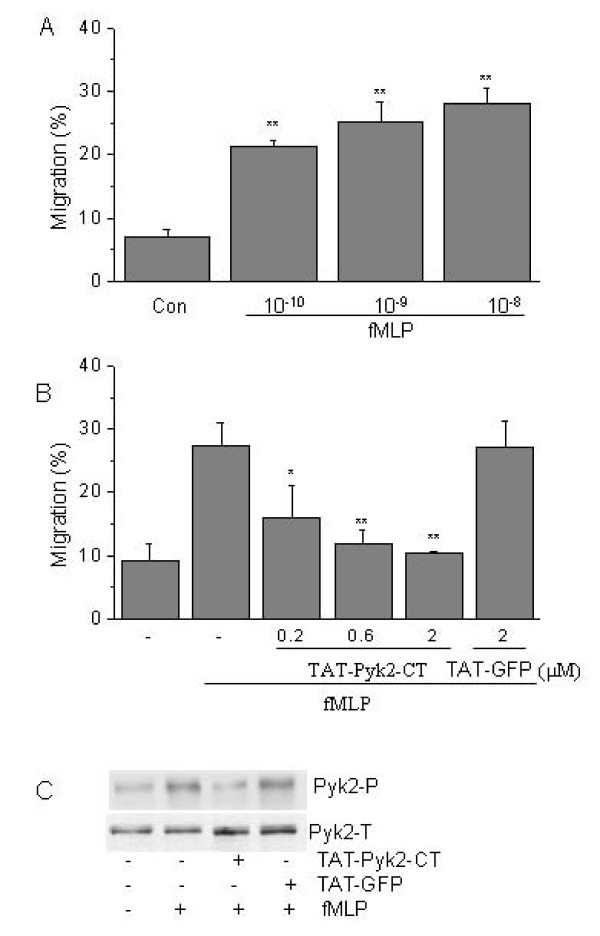
**A) Concentration-dependent effect of fMLP on migration of dHL60 cells through a transmembrane filter**. An increased concentration of fMLP was added to the bottom wells while the dHL60 cells were added on to the top plates. dHL60 cell migration was quantitated by MPO activity of the bottom wells. Results are presented as the mean ± SEM from 4 separate experiments. ** p < 0.01 vs. buffer control by Fisher's LSD test. B) Effect of TAT-Pyk2-CT on chemotaxis of dHL-60 cells to 10^-8 ^M fMLP. dHL-60 cells were preincubated with TAT-Pyk2-CT or TAT-GFP and were added on the top plate of the migration system. dHL-60 cell migration in response to 10^-8 ^M fMLP was measured after 90 min. Results are presented as the mean ± SEM from 4 separate experiments. *P < 0.05 and **p < 0.01 vs. fMLP alone by Fisher's LSD test. C) Effect of TAT-Pyk2-CT on autophosphorylation of Pyk2 induced by fMLP in adherent dHL60 cells. dHL60 cells were preincubated with 2 μM TAT-Pyk2-CT or 2 μM TAT-GFP for 30 min, and then added to Fg-coated plates with 1 μM fMLP for 10 min. Pyk2 phosphorylation was detected by Y402 phosphorylation-specific antibody (upper panel). Total Pyk2 expression was detected by anti-Pyk2 antibody (lower panel). Representative blot is shown (n = 3).

fMLP induced concentration-dependent transwell migration of dHL60 cells (Fig. 3A). Similar to human neutrophils [21], the number of dHL60 cells that migrated through the transwell filter decreased at higher concentrations than 10^-8 ^M due to saturating diffusion of fMLP into the upper chamber. Therefore, 10^-8 ^M fMLP was selected as the optimal concentration for subsequent migration assays.

TAT-Pyk2-CT blocked migration of dHL60 cells in concentration-dependent manner (Fig. 3B). Migration of dHL60 increased from 9.2 ±2.6% to 27.4 ± 3.6% in response to fMLP. TAT-Pyk2-CT, which caused no inhibition of cell adhesion (see above), significantly blocked transwell migration of dHL60 cells. TAT-Pyk2-CT decreased cell migration to 16.0 ± 5.1% at 0.2 μM (p < 0.05) and to 10.4 ± 0.2% at 2 μM (p < 0.01). A control TAT protein, TAT-Green fluorescent protein (TAT-GFP), had no inhibitory effect on migration of dHL60 cells at 2 μM.

To confirm the specificity of inhibition of Pyk2 activation caused by TAT-Pyk2-CT, dHL60 cells were preincubated with 2 μM TAT-Pyk2-CT or TAT-GFP for 30 min and then stimulated with fMLP in Fg-coated plates. TAT-Pyk2-CT inhibited Pyk2 autophosphorylation elicited by fMLP (Fig. 3C). By contrast, TAT-GFP had no effect on Pyk2 phosphorylation caused by fMLP in adherent dHL60 cells.

### Effect of TAT-Pyk2-CT on fMLP-stimulated spreading of dHL60 cell

**Figure 4:**
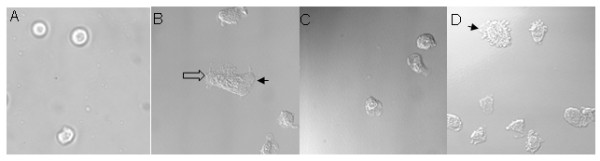
**Effect of Pyk2 inhibition on spreading of dHL60 cells on plated fibrinogen**. dHL-60 cells were preincubated with indicated concentrations of 2 μM TAT-Pyk2-CT (C) or TAT-GFP (D) for 15 min at 37°C, and adhered to plated fibrinogen for 30 min in the absence (A) or presence (B-D) of 1 μM fMLP. Cell morphology was analyzed by light microscopy. Representative images were shown from three experiments. Filled arrow points to lamellipodia, and empty arrow points to filopodia.

Pyk2 was essential for spreading of dHL-60 cells caused by 10^-6 ^M fMLP on Fg-coated plates (Figs 4A and 4B). In the unstimulated control state (Fig. 4A), cells were rounded and non-specifically adherent, which accounts for their brighter appearance. fMLP-stimulated dHL60 cells occupied a larger surface area in contact with the substratum than non-stimulated cells. Phase contrast microscopy revealed dark and irregularly shaped cells after treatment with fMLP compared to controls (Fig. 4B). Spreading of adherent dHL60 cells on Fg coated plates was blocked by dominant negative TAT-Pyk2-CT (Fig. 4C). Phase-contrast microscopy demonstrated only minimal irregularity in dHL-60 cell shape after TAT-Pyk2-CT. Blockade caused by TAT-Pyk2-CT was specific. Pretreatment with TAT-GFP did not block cell spreading (Fig. 4D), and morphology was similar to untreated cells (Fig. 4A).

FMLP also caused formation of filopodia and lamellipodia (Fig. 4B). Formation of these structure also was blocked by TAT-Pyk2-CT (Fig. 4C), but not by TAT-GFP (Fig. 4D).

### Localization of phospho-Pyk2 in dHL60 cells adherent to plated fibrinogen

**Figure 5:**
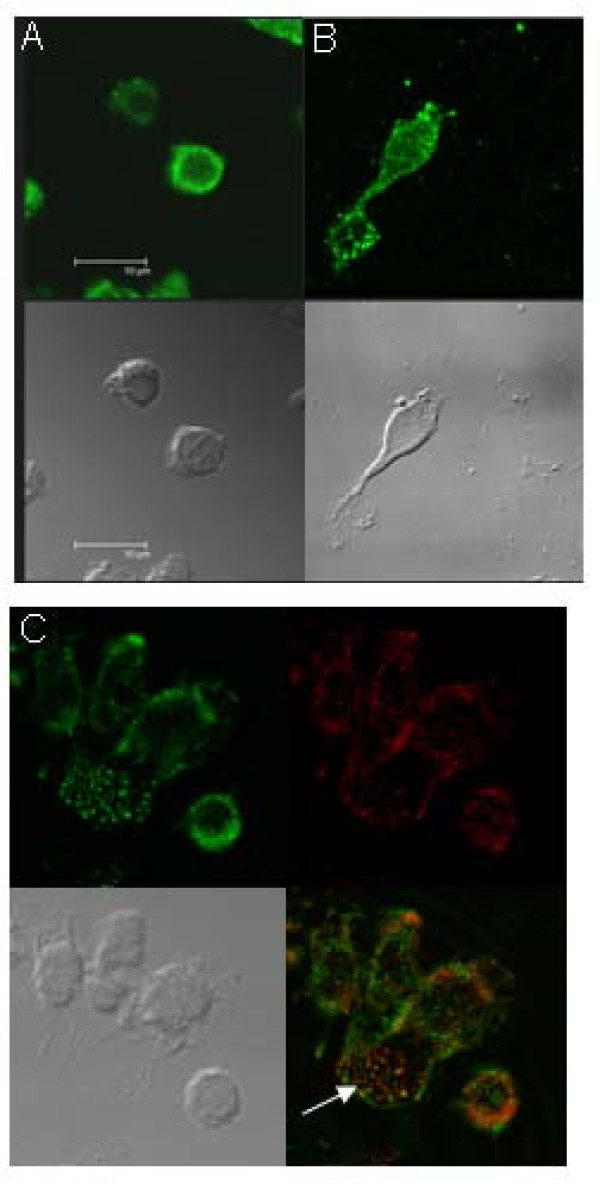
**Localization of Pyk2 in unstimulated and fMLP-stimulated adherent dHL-60 cells**. dHL-60 cells (2 × 10^5^) were added to fibrinogen-coated 8-well chamber slides with or without 1 μM fMLP for 30 min. Pyk2 localization in unstimulated (A) or fMLP-stimulated cells (B-C) was detected by anti-phospho Pyk2 antibody. Phospho-Pyk2 was localized adjacent to cell membrane as a round circle in non-stimulated control cells, while it concentrates on pseudopods and uropods of polarizing cells (B) in a punctate staining after fMLP stimulation. The lower panel of figure A and B were transmission images. C) Colocalization of Pyk2 and F-actin in dHL-60 at lamellipodia. dHL-60 cells (2 × 10^5^) were added to fibrinogen-coated 8-well chamber slides with 1 μM fMLP for 30 min. Pyk2 localization in dHL-60 cells was detected by anti-phospho Pyk2 antibody (Green, upper left). F-actin was detected by Alexa Fluor 647 phalloidin staining (Red, upper right). The transmission image was shown in lower left, and the colocalization of Pyk2 and F-actin was shown in lower right in yellow. Representative images were shown from four experiments. Arrow points the colocalization in lamellipodia.

Pyk2 is tyrosine phosphorylated during cell adhesion [15]. In buffer-treated adherent cells, phospho-Pyk2 was localized uniformly and circumferentially around the cell membrane (Fig. 5A). In cells in which 10^-6 ^M fMLP caused cell spreading, phospho-Pyk2 was distributed almost entirely in the periphery, corresponding to the shape of the cell (Fig. 5B). Phospho-Pyk2 was visualized in highest concentration at the leading edge of spreading HL-60 cells. In fMLP stimulated non-spreading cells, the localization of phospho Pyk2 was similar to the non-stimulated cells (Fig. 5C). Phospho-Pyk2 also co-localized with F-actin at the leading edge of the lamellipodia (Fig. 5C).

### Effect of silencing Pyk2 on dHL60 cell migration

**Figure 6:**
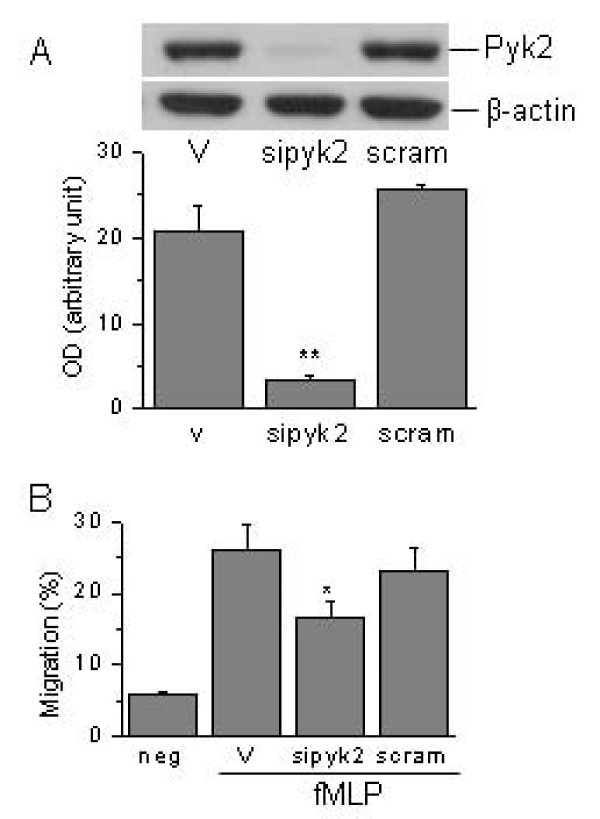
**Effect of silencing Pyk2 expression on chemotaxis of dHL-60 cells to fMLP**. A) Representative Western blot of Pyk2 expression in dHL60 cells (top panel). dHL-60 cells were resuspended in nucleofector solution V (V), and then transfected with 2 μM Pyk2 siRNA (sipyk2) or non-targeting scramble control siRNA (scram). Pyk2 expression in dHL60 cells was measured 3 days after transfection by Western blotting analysis using anti-Pyk2 antibody, and equal protein loading was verified by anti-β actin antibody. The bottom panel was the densitometric analysis of Pyk2 bands from three blots. **P < 0.01 vs. nucleofector solution V vehicle control or scramble control using paired t test. B). Effect of silencing Pyk2 expression on migration of dHL60 cells. Migration assays were performed 72 h after transfection on Transwell plates as above. Migration in response to 10^-8 ^M fMLP was measured after 90 min. Results are presented as the mean ± SEM from 3 separate experiments. *P < 0.05 vs. nucleofector solution V vehicle control or scramble control using paired t test.

Pyk2 siRNA reduced the expression of Pyk2 protein by ~85% as compared to vehicle control (Nucleofector Solution V only), analyzed by Western blot and densitometry. By contrast, the scramble siRNA control had no effect on Pyk2 protein expression (Fig. 6A).

Silencing of Pyk2 expression significantly reduced the migration of dHL60 cells compared to cells treated with Nucleofector Solution V vehicle control or scramble siRNA control. Pyk2 siRNA reduced migration of dHL60 cells to fMLP from 25.9 ± 3.8% of vehicle control to 16.6 ± 2.2% (p < 0.05, vs. vehicle control or scramble siRNA). The scramble non-targeting siRNA had no effect on dHL60 cell migration to fMLP (NS vs. vehicle control). Pyk2 siRNA had no effect on dHL60 cells differentiation as measured by nuclear morphology and CD11b surface expression (data not shown).

## Discussion

The objective of this investigation was to determine the role of Pyk2 in dHL60 cell migration. We found that 1) Pyk2, especially the hematopoietic form of Pyk2, is induced after neutrophilic differentiation and is tyrosine phosphorylated upon adhesion to plated fibrinogen, 2) Pyk2 does not regulate β_2_-integrin adhesion of dHL60 cells to fibrinogen, as inhibition of Pyk2 by TAT-Pyk2-CT does not block cell adhesion, 3) Pyk2 is essential for dHL60 migration, as both inhibition of Pyk2 activity or silencing of Pyk2 expression reduced the migration of dHL60 cells, and 4) Pyk2 is concentrated to the cell membrane extensions in migrating dHL60 cells.

Pyk2 is a non-receptor protein tyrosine kinase that is closely related to the p125 focal adhesion kinase (Fak). Here we report that both forms of Pyk2 are induced in DMSO-differentiated cells, and the alternatively spliced, hematopoietic form of Pyk2 (Pyk2H) is the predominant isoform expressed in dHL60 cells. Pyk2-H is mainly expressed in hematopoietic cells including T-cells, B-cells, and natural killer cells [22] and is also expressed in CD34+ bone marrow progenitor cells and myeloid leukemic cells [23,24]. The two Pyk2 isoforms appear functionally redundant for inhibiting integrin-mediated myelopoiesis when singly overexpressed in primary CD34^+ ^cells [24]. Stimulation of dHL60 cells with fMLP in fibrinogen-coated plates induced the tyrosine phosphorylation of both forms of Pyk2 (Fig. 2A, 2B), while minimal phosphorylation is detected in suspension cells. Adhesion of dHL60 cells to plated Fg is mediated by β_2_-integrin; as preincubation with anti-CD18 antibody blocked dHL60 cell adhesion to plated fibrinogen. Previous studies have suggested that Pyk2 is involved in the functional activation of dHL60 cells in two signaling pathways: an fMLP receptor-mediated "inside-out" signaling pathway that can cause β2 integrin activation and a subsequent β2 integrin-mediated "outside-in" signaling pathway [19], although the functional activation of integrin was not determined in that study. Our results indicate that Pyk2 is not involved in fMLP induced inside-out signaling for increased adhesiveness of β2 integrin, as Pyk2 inhibition by TAT-Pyk2-CT had no inhibitory effect on adhesion of dHL60 cells to plated fibrinogen (Fig. 2D). We also found that preincubation of dHL60 cells with anti-CD18 blocks both cell adhesion and Pyk2 phosphorylation (Fig. 2B, 2C). These results indicate that tyrosine phosphorylation of Pyk2 is activated after β_2_-integrin binding to plated Fg and is required for β2 integrin mediated outside-in signaling for subsequent cell spreading and migration (see below).

Integrin-mediated adhesive interactions with substrate proteins play a critical mechanical role in the morphologic change of cells and migration by linking the extracellular matrix proteins to the intracellular cytoskeleton. In this study, Pyk2 inhibition by TAT-Pyk2-CT dramatically reduced the extension of cell membranes on Fg-coated plates and the formation of large lamellipodia (Fig. 4C), suggesting that Pyk2 activation plays a key role in the morphological change following cell adhesion. The extension of lamellipodia, filopodia and other membrane processes depends on reorganization of the actin cytoskeleton as well as the formation of new actin polymers that support membrane protrusions [25]. We found that Pyk2 and F-actin are colocalized and concentrated in lamellipodia of migrating dHL60 cells (Fig. 5C). Furthermore, Pyk2 inhibition by TAT-Pyk2-CT prevented the formation of large lamellipodia (Fig. 4C). These results suggest that integrin-mediated Pyk2 activation may play a role in cytoskeletal reorganization during dHL60 cell spreading and migration.

Cell spreading and the extension of membrane processes play an essential role in cell migration. During chemoattractant-induced neutrophil migration, cells assume a polarized morphology and then spread and extend membrane processes in the direction of movement as they move along extracellular matrix components in response to a gradient of chemoattractant. Integrin-mediated adhesion at the leading edge of the migrating cell provides the traction necessary for forward movement [26]. Such directional movement is essential for neutrophils to enter into sites of infection to fight bacteria. Our findings suggest that Pyk2 may regulate integrin-mediated extension of membrane processes and provide a possible mechanism by which Pyk2 promotes dHL60 cell migration (Fig. 3).

It is important to note that Pyk2 knockdown in dHL60 cells (~85%) only blocked the chemotaxis of dHL60 cells by ~46%. One explanation for the incomplete inhibition of migration is that the residual Pyk2 expression after knockdown is sufficient to enable some chemotaxis. Another possibility is that multiple parallel pathways exist in cells that enable cell chemotaxis. It has been found that four independent signaling pathways lead to the chemotaxis of Dictyostelium discoideum cells; these are phosphoinositide-3 kinase, phospholipase A2, soluble guanylyl cyclase, and cGMP [27]. Similarly in human neutrophils, both phosphoinositide-3 kinase and p38 MAP kinase regulate chemotaxis in a fMLP gradient [28,29]. A recent study in dHL60 cells found that phosphoinositide-3 kinase and Src pathways work in parallel to modulate chemotaxis to IL-8 [20]. Other studies have found that Pyk2 is the upstream kinase for Src activation [30,31]. Therefore, it is reasonable to speculate that Pyk2 knockdown in dHL60 cells impairs only the Src-mediated dHL60 chemotaxis pathway, while other parallel pathways may enable some residual chemotaxis capability as observed in this study.

It is important to recognize some other limitations of our findings. We used the differentiated HL-60 cells to model human neutrophils as these dHL60 cells can be modified genetically. Although the differentiated HL-60 cells have many features that mimic mature human neutrophils [13,32,33], such as CD11b/CD18 expression, responsiveness to fMLP and other chemoattractants, have a segmented nucleus, these cells are nevertheless far-different from mature neutrophils, as they lack the ability to synthesize granular proteins as well as to form granules [34]. Cell adhesion and migration occurred only in 20-30% of cells. This fraction is however similar to that for mature PMN [35]. This likely represents a limitation of the adhesion and migration apparatuses rather than heterogeneity in dHL60 and PMN cells. Additionally, the isoform of Pyk2 expressed in dHL60 cells is different from that of mature neutrophils, with dHL60 predominantly expressing the hematopoietic form while mature neutrophils expressed the full-length form. However, no functional difference between these two isoforms has been elucidated previously. The use of dHL60 cells was essential because a relative long-lived cell model was required for these siRNA silencing studies.

## Conclusions

In summary, we find that β_2_-integrin adhesion of dHL60 cells induces activation of Pyk2. Specific inhibition of Pyk2 by TAT-Pyk2-CT causes a substantial attenuation of dHL60 cell spreading and migration, but has no effect on cell adhesion, suggesting a critical role for Pyk2 in morphological change and migration of neutrophilic dHL60 cells.

## Abbreviations

dHL-60: Differentiated HL-60 cells; BSA: Bovine serum albumin; DMSO: Dimethyl sulfoxide; FAK: Focal adhesion kinase; Fg: Fibrinogen; fMLP: formyl-Met-Leu-Phe; FBS: Fetal bovine serum; GFP: Green fluorescent protein; HBSS: Hanks balanced salt solution; MPO: Myeloperoxidase; Pyk2: Proline-rich tyrosine kinase; Pyk2H: Hematopoietic cell-specific Pyk2; PMN: Polymorphonuclear neutrophils; siRNA: Small interfering RNA.

## Competing interests

The authors declare that they have no competing interests.

## Authors' contributions

LW carried out the cell culture of HL60 cells, adhesion and migration assays, data analysis and prepared the first draft of the manuscript. JL performed the western blot for Pyk2 expression, siRNA transfection and migration experiments. YD performed spreading assay and confocal microscopy experiments. AL co-developed the study idea, participated in the design of the study and the final preparation of the manuscript. XZ developed the study idea, designed and coordinated the experimental work and spearheaded the final preparation of the manuscript. All authors read and approved the final manuscript.
